# Dental Infection Causing Methicillin-Resistant Staphylococcus aureus Bacteremia and Spinal Infection: A Case Report

**DOI:** 10.7759/cureus.51136

**Published:** 2023-12-26

**Authors:** Nicholas R Munoz, Ali Ghorbani, Chibuike C Agwuegbo, John M Vincent Coralde

**Affiliations:** 1 Internal Medicine, Southwest Healthcare, Temecula, USA

**Keywords:** methicillin-resistant staphylococcus aureus (mrsa), methicillin-resistant staphylococcus aureus bacteremia, periodontal disease (pd), drug use, infectious disease medicine, crohn’s disease (cd), vertebral discitis, dental infection, spinal abscess

## Abstract

Poor dentition as a source of infection causing bacteremia and spinal infections (such as paraspinal abscess, and discitis) should be considered even in the absence of recent dental surgery. The case presents a patient found to have an infection in the cervical and thoracic spine, methicillin-resistant *Staphylococcus aureus* bacteremia, and poor dentition. Although the patient had a history of drug use, he denied a history of intravenous drug use. He had Crohn’s disease that resulted in periodontal and endodontal tooth disease. The patient was found to have poor dentition with erythematous gums. He had not been to the dentist in over 24 years and had active dental caries. Since he presented with bacteremia and a spinal infection, it is likely the patient had an infection in the oral cavity that spread hematogenously to the blood, and then the spine. This report highlights the importance of considering tooth infections as the source of bacteremia and spinal infections.

## Introduction

Crohn’s disease is a gastrointestinal disease marked by chronic inflammation of the bowel. It has been linked to dental disease, as it can cause salivary dysfunction leading to xerostomia and tooth decay [1]. Odontogenic infections have been shown to lead to sepsis, and even death if untreated [2]. Infections that start in the oral cavity can spread deeper through tissues into the retropharyngeal space, as well as hematogenously through the blood [3-5]. Many infections in the head and neck region are odontogenic in nature, rarely these infections can lead to sepsis [6]. In one study with 483 patients who were hospitalized with severe odontogenic infections, only 3.3% were found to be septic [7]. Odontogenic infection followed by prompt treatment has been shown to improve outcomes such as decreased mortality [2]. Spinal infections such as septic discitis, often present with neurological symptoms, fever, and back pain. The discovery of a causative organism is crucial as patients without isolation of a causative organism have been found to have poorer outcomes such as increased mortality [8]. The case presents a patient with a history of Crohn’s disease who complained of fevers, chills, and back pain. He was found to have a paraspinal abscess, discitis, arachnoiditis, methicillin-resistant *Staphylococcus aureus* (MRSA) bacteremia, periodontal, and tooth disease. It is likely bacteria from the patient's oral cavity spread hematogenously causing MRSA bacteremia and a spinal infection.

## Case presentation

The patient is a 54-year-old male with a past medical history of polysubstance abuse, Crohn's disease, asthma, gout, and arthritis who presented to the hospital with severe neck pain and back pain. At that time, he received a CT scan of the spine which showed no evidence of infection. Blood cultures were drawn as he had an elevated white blood cell count. He was discharged with muscle relaxers and instructions to follow up with his primary care physician. Several days later the blood cultures resulted as positive for MRSA bacteremia. The patient was called and instructed to return to the hospital.

When the patient returned to the hospital, he admitted to having fevers and chills along with severe back pain. Additionally, he endorsed numbness and tingling of the right foot which radiated to the proximal right thigh as well as neck stiffness that resolved a week previously. The patient was having difficulty ambulating due to back pain and foot neuropathy. He stated he had chronic tooth pain since he was in his 20s and had not seen a dentist for over 24 years. In the month before his hospitalization, two of his molar teeth had fallen out. After his teeth fell out he had increasing pain on the left side of the jaw which radiated to the back of the neck. The pain slowly migrated from the back of the neck to the lower back impairing his ability to work as his job required heavy lifting. The patient denied sick contacts, traveling, trauma, dizziness, saddle anesthesia, urinary incontinence, fecal incontinence, light sensitivity, neck pain, and headache. He stated he had a history of heroin abuse although he quit over one year ago. He would previously smoke heroin, although denied a history of injecting drugs intravenously. He said that recently due to his back pain, he started taking oxycodone, methamphetamine, and fentanyl orally.

He presented with a blood pressure of 155/97 mmHg, a heart rate of 107 beats per minute, a respiratory rate of 18 breaths per minute, and a blood oxygen saturation of 97%. The patient was intermittently febrile with temperatures up to 39.1°C. His heart had a regular rate and rhythm, his lungs were clear to auscultation bilaterally, and he had multiple missing teeth with poor dentition and erythematous gums. No purulence or bleeding of the gums was observed. The patient had lower back pain on elevation of the right leg and tenderness to palpation of the right and left flanks. He denied tenderness on palpation of the cervical, thoracic, lumbar, and sacral spine. He had a full range of motion of the neck and denied neck pain during active movement. He had negative Kernig's and Babinski's signs, with normal strength of the upper and lower extremities bilaterally. His cranial nerves were intact. His labs were positive for leukocytosis up to 16,600 cells/mcL (Table 1). Urine drug screen was positive for amphetamines, fentanyl, and opioids. During his hospitalization, he received five sets of blood cultures which were all positive for MRSA, resistant to oxacillin, and sensitive to vancomycin.

**Table 1:** The patient's laboratory values on admission The star (*) icon indicates an abnormal lab value.

	Patients lab value	Reference range
White blood cells	16,200 cells/uL*	4,5000-11,000 cells/uL
Hemoglobin	12.6 g/dL*	13.2-16.6 g/dL
Platelet	164,000 platelets/uL	150,000 platelets/uL
Sedimentation rate	99 mm/hr*	0-32 mm/hr
C-reactive protein	>75.0 mg/L*	<3 mg/L
Glucose level	119 mg/dL*	70-100 mg/dL
Sodium	134 mmol/L*	135-145 mmol/L
Potassium	3.5 mmol/L	3.5-5.0 mmol/L
Chloride	96 mmol/L	96-106 mmol/L
CO_2_	30 mmol/L	21-32 mmol/L
Blood urea nitrogen	16 mg/dL	7-18 mg/dL
Creatinine	1.0 mg/dL	0.7-1.3 mg/dL
Lactic acid	1.2 mmol/L	0.4-2.0 mmol/L

**Figure 1:**
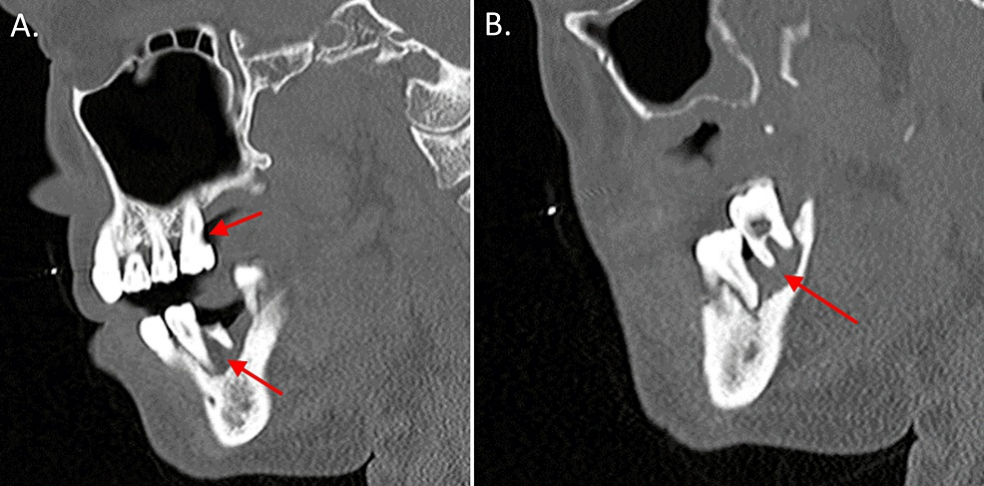
(A) CT scan of the maxillofacial area with contrast showing a sagittal view of the patient's endodontal and periodontal disease of the right teeth (arrows). (B) CT scan of the maxillofacial area with contrast showing a sagittal view of the patient's periodontal disease of the left teeth (arrow)

**Figure 2:**
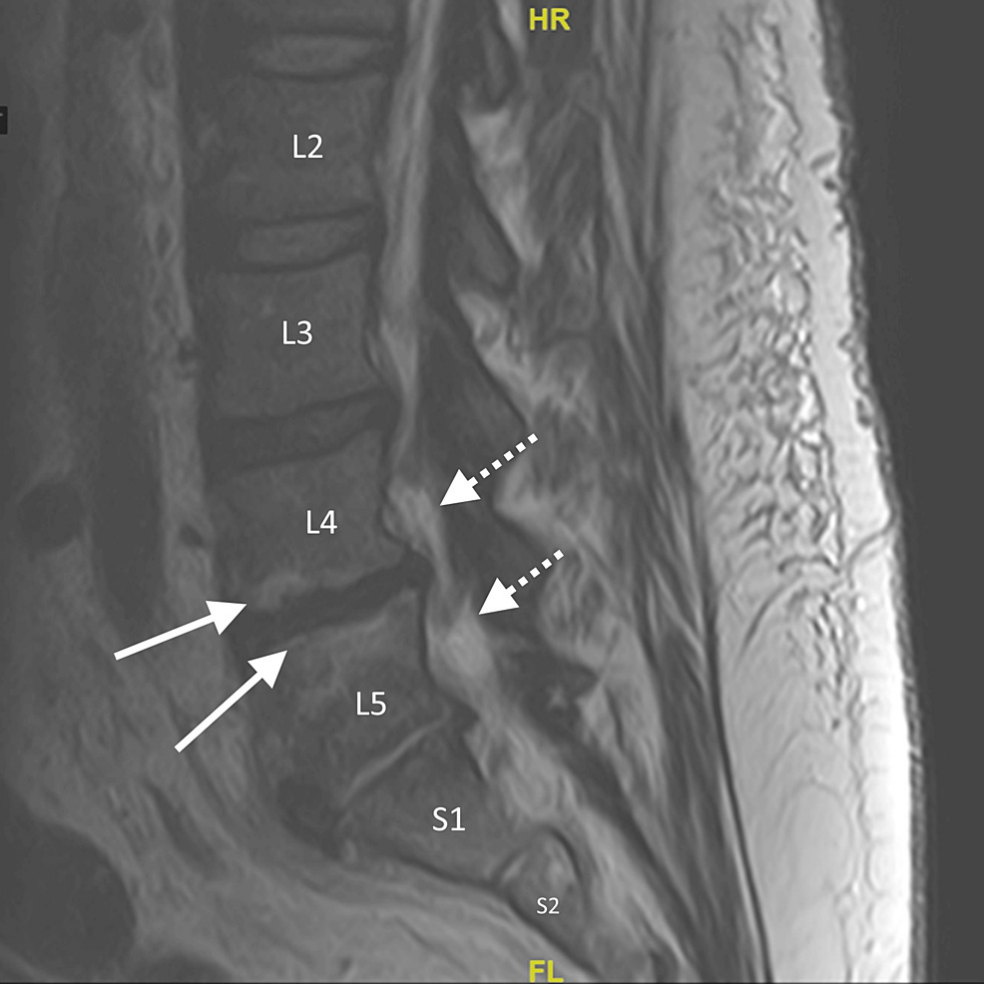
MRI of the patient's lumbar spine with and without contrast showing discitis at L4-L5 (solid arrows) with abnormal enhancement of the anterior paraspinal soft tissues from L4-S2 with enhancement and thickening of the caudal thecal sac and distal roots of the cauda equina likely representing arachnoiditis (dotted arrows)

**Figure 3:**
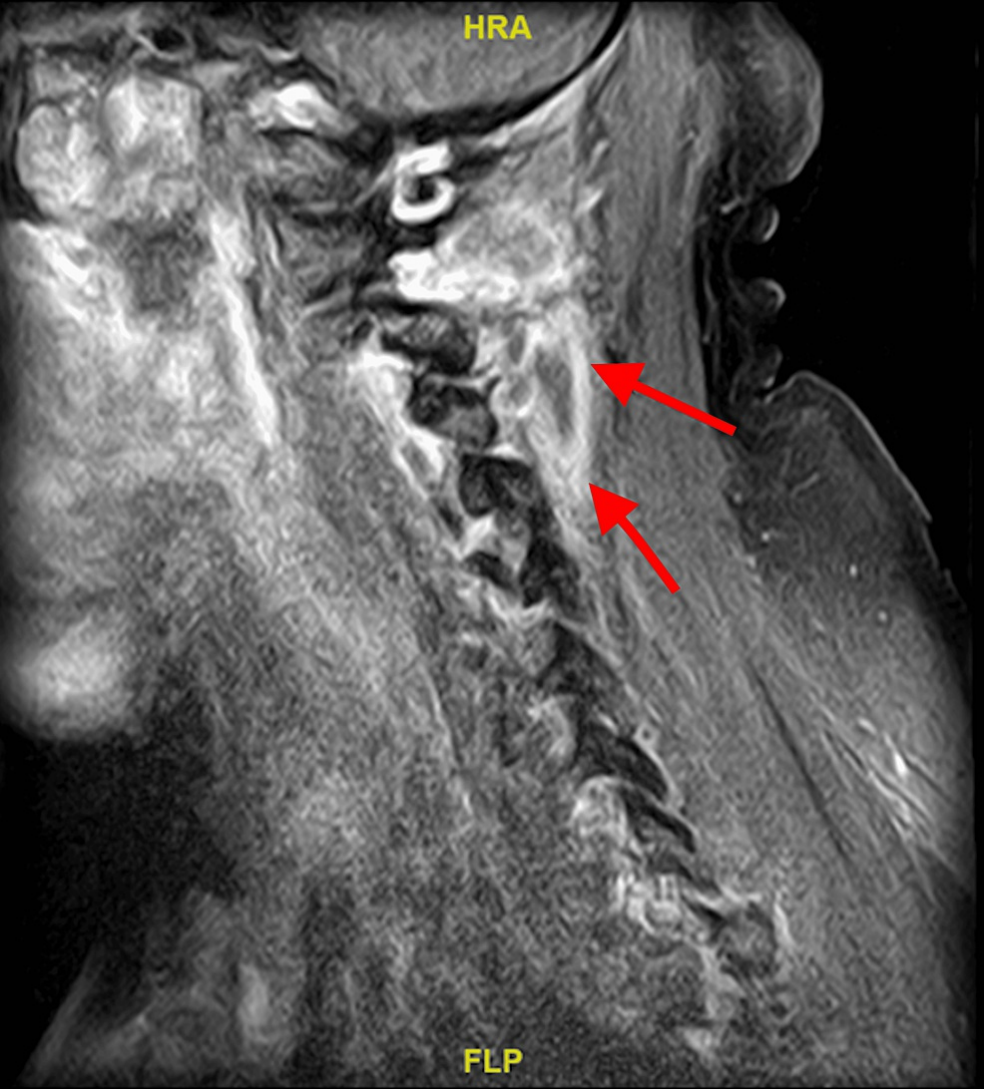
MRI with contrast of the patient's cervical spine showing a soft tissue lesion likely reflecting abscess with surrounding edema and enhancement (arrows)

The patient received a CT scan of the maxillofacial area with contrast showing endodontal and periodontal disease involving the molars (Figure 1). He received an MRI of the lumbar spine with and without contrast that showed discitis at L4-L5 with abnormal enhancement of anterior paraspinal soft tissues from L4-S2, as well as enhancement and thickening of the caudal thecal sac and distal roots of the cauda equina likely representing arachnoiditis (Figure 2). MRI of the thoracic spine with contrast showed no evidence of pathological enhancement. MRI of the cervical spine showed a soft tissue lesion likely reflecting an abscess at the C3 level with surrounding edema and enhancement. There was no evidence of osteomyelitis, discitis, or epidural abscess in the cervical spine (Figure 3).

The patient received a transesophageal echocardiogram which showed an ejection fraction of approximately 60% and no evidence of aortic, mitral, pulmonic, or tricuspid valve vegetations. The patient was initially started on intravenous (IV) vancomycin, ceftriaxone, ampicillin, and metronidazole. After the patient's workup was completed, the differential diagnosis was narrowed to arachnoiditis, periodontal infection, endodontal infection, bacteremia, and paraspinal abscess. The infectious disease team was consulted and recommended downgrading antibiotics to vancomycin only. Neurosurgery was consulted and stated no surgical intervention or lumbar puncture was necessary. Neurology was consulted and advised by the infectious disease team that a lumbar puncture was not necessary as there was a high probability that the MRSA in the patient's blood was the causative organism of his spinal pathology. His fever resolved and his pain improved with treatment. He had been receiving IV antibiotics for a few days when he was found to have a lighter and a bag of white powder that was thought to be illegal drugs. When hospital security called the Sheriff's Department, the patient eloped against medical advice.

## Discussion

Spinal infections such as septic discitis can present with symptoms such as back pain, weight loss, sensory loss, radiculopathy, weakness, urinary retention, and hyporeflexia [8]. Patients commonly present with infectious symptoms such as fever, night sweats, leukocytosis, and back pain [8]. Diagnosis is often made by MRI although CT scanning may also play a role [8]. Common organisms implicated in discitis are *Escherichia coli, S. aureus, Streptococcus pneumoniae*, and gram-negative bacteria [8]. Early vertebral osteomyelitis and discitis can be seen on MRI as subtle endplate edema in the presence of disc degeneration and two contiguous vertebrae with inflammatory changes seen in the intervertebral disc. These features can be seen initially although they usually take time to develop [9]. The Delmarter classification scheme is used to classify the imaging characteristics of arachnoiditis. It includes an adherent mass of centrally located nerve roots in the thecal sac, an empty thecal sac with peripherally adherent nerve roots, and a signal mass of soft tissue obliterating or replacing the subarachnoid space [10]. Paraspinal abscesses are often seen as inflammatory lesions adjacent to the spinal cord [11].

*S. aureus* is one of the most common causes of bacteremia in advanced industrialized nations [12]. MRSA bacteremia has a higher mortality rate than methicillin-sensitive *S. aureus*. It is associated with a large range of infections such as infective endocarditis, soft tissue, pulmonary, device-related, and osteoarticular infections [13]. MRSA bacteremia has been associated with oral infections. Even a mild infection of the gums can lead to the formation of an abscess that could expand to the paratracheal and neck spaces. Surgical debridement and drainage is the recommended action for odontogenic infections to remove the source of infection and prevent spread to other anatomical spaces [2]. Most infections of the head and neck are odontogenic. They can lead to complications such as sepsis, necrosis, endocarditis, and compromised airways. Factors that can exacerbate the severity of dental infections include diabetes, obesity, immunodeficiency, alcohol use, and autoimmune disorders such as lupus. One study showed a 3.3% rate of sepsis in patients with tooth infections over an observation period of five years, these patients had at least one comorbidity or risk factor for severe disease [7].

**Figure 4:**
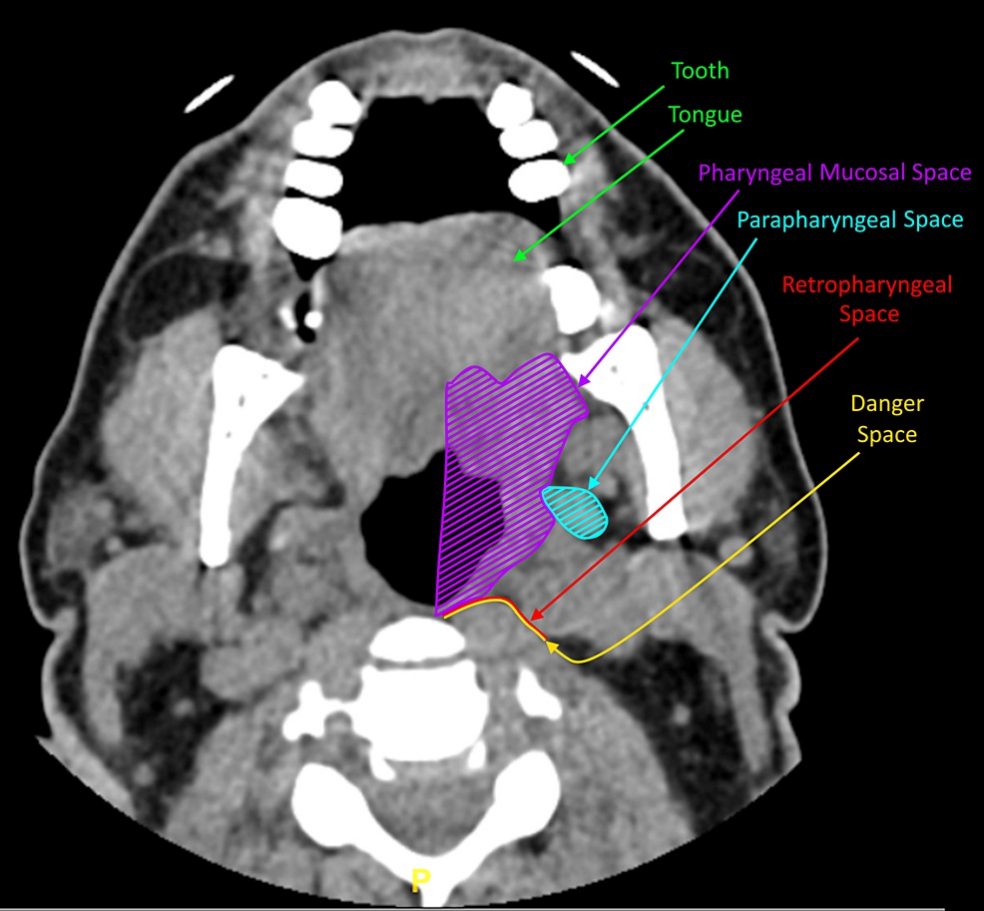
Transverse view of a CT scan with contrast of the patient's head Arrows indicate pertinent anatomical landmarks that infection may travel when spreading contiguously from the oral cavity into deeper tissue spaces.

**Figure 5:**
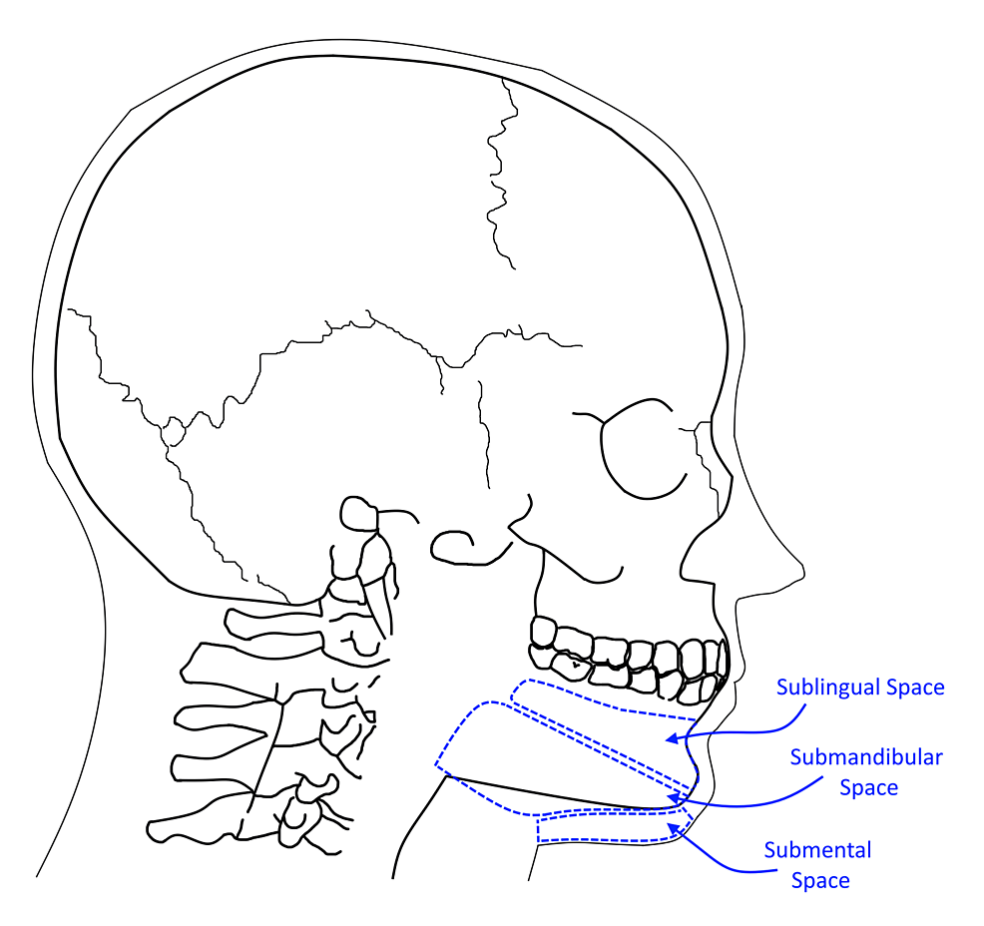
An illustration indicating the sublingual, submandibular, and submental spaces Image Credits: Nicholas R. Munoz

Infection from the posterior teeth and periodontal tissue, mainly those above the mylohyoid line, can spread deeper to the parapharyngeal and retropharyngeal space. From there, the infection may further spread to the danger space and the mediastinum (Figure 4). Furthermore, the infection can spread laterally and access paraspinal spaces. Infections of the mandibular teeth can spread contiguously in different ways including the sublingual, submandibular, and submental spaces (Figure 5). The spread of odontogenic infections of the mandible is largely based on the position of the tooth roots or periodontal infection relative to the mylohyoid line. The mylohyoid inserts on the superior surface of the hyoid bone and originates from the lingual cortex of the mandibular body at a point known as the mylohyoid line, which is an oblique line that slopes inferiorly from posterior to anterior. Infection spreading medially and inferiorly along dental roots superior to this line will result in infections of the sublingual space; spread along those roots inferior to the line will result in submandibular space infections [3,4]. It is possible the patient’s infection could have spread continuously through tissue spaces into the spine, although in this case, it is more likely the infection spread hematogenously from the teeth to the spine. On imaging the patient had no evidence of inflammation or abscesses in the head or neck spaces posteriorly to the teeth. Additionally, the patient presented with MRSA bacteremia.

Paraspinal abscesses and infectious discitis are often successfully managed conservatively. Conservative management consists of IV antibiotics, pain medications, spinal brace immobilization, physical therapy, and bed rest. Bed rest for a period of two to six weeks is recommended depending on the site of infection, the extent of deformity, the age of the patient, and risk factors for complications. Antibiotic penetration has been shown to be better in the paraspinal tissues than in non-vascularized intervertebral disc space [14]. Antibiotic therapy begins with empiric broad-spectrum coverage with fluoroquinolones or a third-generation cephalosporin in combination with clindamycin or vancomycin. The regimen is subsequently narrowed down based on the organism isolated in the culture. If there is a concern for mycobacterial or fungal etiology, appropriate anti-mycobacterial and anti-fungal agents should be initiated. If patients fail to improve despite adequate conservative treatment for four weeks or if significant progression of neurological deficits with destruction of vertebrae is noticed, conservative management should be discontinued and surgical treatment initiated. Surgery provides a more rapid cure with debridement and removal of the septic foci as well as stabilization of the affected segment with laminectomy and subsequent bony fusion [15]. Specimens should also be obtained during surgery for microbiological testing. If osteosynthetic material is used, an antibiotic carrier should be inserted to prevent infection of prosthetic material [16].

Crohn's disease is a chronic inflammatory bowel condition that has been linked with an increased risk of dental caries along with periodontal disease which can subsequently lead to bacteremia. Multiple interconnected factors contribute to the association of Crohn’s disease and bacteremia. Individuals with Crohn's disease often experience nutrient malabsorption resulting in multiple nutritional deficiencies. This can impact their overall oral health as insufficient intake of essential minerals and vitamins can lead to enamel erosion and weaker teeth, making them more susceptible to dental caries. Additionally, Individuals with Crohn's disease can have an increased rate of xerostomia. Reduced saliva production, limits the mouth's natural defense mechanism against harmful bacteria increasing the risk of dental caries [1]. This imbalance in the oral flora can lead to the growth of virulent bacteria and further increase the susceptibility to tooth decay [17].

Periodontal disease, an inflammatory condition affecting the supporting structures of the teeth, has also been linked to bacteremia. This association arises due to the compromised oral health associated with periodontal disease. As periodontal disease progresses, the chronic inflammation and bacterial biofilms in the gums can yield a pathway for bacteria to enter the bloodstream. During activities like toothbrushing or dental procedures, the disruption of the oral tissues can release oral bacteria into the bloodstream, potentially leading to bacteremia. This condition is of particular concern because it can result in the translocation of bacteria to various parts of the body, which may contribute to systemic infections and inflammation and has been linked with an increased risk of certain health conditions [18].

In a recent study, the risk of bacteremia associated with routine dental activities like toothbrushing and dental extractions was investigated. The research highlights the potential for periodontal disease and dental procedures to lead to bacteremia, emphasizing the importance of maintaining good oral health and promptly treating periodontal infections to decrease the risk of systemic infections like bacteremia. This study underscores the critical need for both dental professionals and patients to be aware of the systemic implications of periodontal disease and the significance of timely treatment and maintenance of oral health [18].

In the case presented the patient had a history of non-IV drug use as well as significant endodontal and periodontal disease caused by ulcerative colitis. He presented with spinal infections in the cervical and thoracic spine. Although he denied intravenous drug use, it is likely his substance abuse contributed to his infection by decreasing his pain making it easier for him to delay seeking medical attention. It is likely the source of his infection was the oral cavity. Infections from the oral cavity can spread to the spine either through contiguous tissue spaces or hematogenously through the blood. It is possible the patient's infection spread through contiguous tissue spaces to the spine although it is more likely the infection spread hematogenously from the oral cavity to the blood, and then to the spine as the patient had MRSA bacteremia.

## Conclusions

Spinal column infections can be either bacterial or fungal. Infections from the oral cavity can seed the spine either hematogenously or contiguously through tissue spaces. In the patient presented it is likely a dental infection spread hematogenously to the blood and then to his spine. In any infection, especially infections of the spine, it is important to find the source of infection. Periodontal and endodontal infection should be considered as a route of infection in patients with spinal infections and bacteremia.
